# The influence of climatic conditions on the transmission dynamics of the 2009 A/H1N1 influenza pandemic in Chile

**DOI:** 10.1186/1471-2334-12-298

**Published:** 2012-11-13

**Authors:** Gerardo Chowell, Sherry Towers, Cécile Viboud, Rodrigo Fuentes, Viviana Sotomayor, Lone Simonsen, Mark A Miller, Mauricio Lima, Claudia Villarroel, Monica Chiu, Jose E Villarroel, Andrea Olea

**Affiliations:** 1 Mathematical, Computational & Modeling Sciences Center, School of Human Evolution and Social Change, Arizona State University, Tempe, AZ, USA; 2 Division of Epidemiology and Population Studies, Fogarty International Center, National Institutes of Health, Bethesda, MD, USA; 3 Department of Mathematics, Purdue University, West Lafayette, IN, USA; 4 Department of Epidemiology, Ministerio de Salud, Santiago, Chile; 5 Department of Global Health, School of Public Health and Health Services, George Washington University, Washington DC, USA; 6 Center for Advanced Studies in Ecology and Biodiversity, Pontificia Universidad Católica de Chile, Santiago, Chile; 7 Applied Meteorology, Dirección Meteorológica de Chile, Santiago, Chile

**Keywords:** A/H1N1 influenza pandemic, Acute respiratory infection, Influenza-like-illness, Reproduction number, Spatial heterogeneity, School cycles, Climatological variables, Specific humidity, Temperature, Chile

## Abstract

**Background:**

The role of demographic factors, climatic conditions, school cycles, and connectivity patterns in shaping the spatio-temporal dynamics of pandemic influenza is not clearly understood. Here we analyzed the spatial, age and temporal evolution of the 2009 A/H1N1 influenza pandemic in Chile, a southern hemisphere country covering a long and narrow strip comprising latitudes 17°S to 56°S.

**Methods:**

We analyzed the dissemination patterns of the 2009 A/H1N1 pandemic across 15 regions of Chile based on daily hospitalizations for severe acute respiratory disease and laboratory confirmed A/H1N1 influenza infection from 01-May to 31-December, 2009. We explored the association between timing of pandemic onset and peak pandemic activity and several geographical and demographic indicators, school vacations, climatic factors, and international passengers. We also estimated the reproduction number (R) based on the growth rate of the exponential pandemic phase by date of symptoms onset, estimated using maximum likelihood methods.

**Results:**

While earlier pandemic onset was associated with larger population size, there was no association with connectivity, demographic, school or climatic factors. In contrast, there was a latitudinal gradient in peak pandemic timing, representing a 16-39-day lag in disease activity from the southern regions relative to the northernmost region (P < 0.001). Geographical differences in latitude of Chilean regions, maximum temperature and specific humidity explained 68.5% of the variability in peak timing (P = 0.01). In addition, there was a decreasing gradient in reproduction number from south to north Chile (P < 0.0001). The regional mean R estimates were 1.6-2.0, 1.3-1.5, and 1.2-1.3 for southern, central and northern regions, respectively, which were not affected by the winter vacation period.

**Conclusions:**

There was a lag in the period of most intense 2009 pandemic influenza activity following a South to North traveling pattern across regions of Chile, significantly associated with geographical differences in minimum temperature and specific humidity. The latitudinal gradient in timing of pandemic activity was accompanied by a gradient in reproduction number (P < 0.0001). Intensified surveillance strategies in colder and drier southern regions could lead to earlier detection of pandemic influenza viruses and improved control outcomes.

## Background

Increasing our understanding of host, environmental, and pathogen specific factors modulating the transmissibility and spatio-temporal dynamics of pandemic influenza has the potential to guide mitigation and surveillance strategies. Several factors have been put forward as potential drivers of the spatio-temporal dynamics of influenza pandemics, including population contact rates, travel patterns, climatic conditions, and geography. Recent studies of the 2009 A/H1N1 influenza pandemic have quantified the role of demographic factors, school cycles and social distancing measures (e.g., school closure) on disease transmission [1-5], but their effect combined with climatic factors is less clear [6,7]. In particular, experimental studies indicate that aerosol transmission of 2009 A/H1N1 influenza is sensitive to temperature and humidity levels [8]. Moreover, the timing and intensity of the 2009 A/H1N1 pandemic waves varied substantially across regions of the world [1,2,9-14], suggesting a potential link with local meteorological conditions. Further, the occurrence of recrudescent waves of pandemic activity in the South-Eastern US in winter 2010 was associated with low humidity levels [15].

The first cases of 2009 A/H1N1 pandemic influenza were confirmed on April 21–23 in California, USA, and Mexico [16,17], and soon after the pandemic virus became the dominant respiratory virus in the Southern Hemisphere’s temperate countries [18,19]. Timing of pandemic activity coincided with the typical influenza winter season in this region which spans the months of May-September. Few studies have characterized A/H1N1 transmission dynamics in the Southern Hemisphere [2,5,20], and no study has explored variation in disease transmission at a small spatial scale in this region. Here we characterized the spatio-temporal dynamics of the 2009 influenza pandemic across Chile, a country which combines an extended latitudinal range with a solid epidemiological surveillance system. In this work, we assessed the relative contribution of environmental conditions, demographic factors, contact rates, and international travel patterns on pandemic A/H1N1 transmission, by modeling a large dataset of weekly hospitalizations for severe acute respiratory infection and laboratory-confirmed A/H1N1 influenza infection in the 15 administrative regions of Chile.

## Methods

### Geographical context

Chile is a South American country covering a long and narrow strip between the Andes mountains to the east and the Pacific Ocean to the west, ranging between latitude 17° and 56°S and longitudes 66° and 76°W. Chile’s climate ranges from dry and desert in the north, Mediterranean in the centre, and rainy temperate in the south. The population of Chile is about 17 million with 40% of the population concentrated in the metropolitan region that includes the Capital, Santiago. Chile is divided into 15 contiguous administrative regions.

### Epidemiological data

We relied on a large individual-level dataset comprising all hospitalizations for severe acute respiratory infection (hereafter referred to as SARI) reported by all public and private hospitals to the Chilean Ministry of Health during 01-May to 31-December 2009. The SARI symptom definition for children < 5 years included pneumonia or severe pneumonia together with any of the following symptoms: hypoxemia, dehydration or loss of appetite, respiratory difficulty, or hemodynamic compromise. The SARI definition for older individuals included any of the following symptoms: tachypnea, hypotension, dyspnea, cyanosis or hypoxemia [21]. The case definition did not change throughout the pandemic period.

A total of 6146 SARI hospitalizations were reported to the Chilean Ministry of Health from May 1 to December 31, 2009, of which 3373 were laboratory tested (54.9%) via reverse transcriptase polymerase chain reaction (RT-PCR), performed by the Instituto Nacional de Salud Pública de Chile (ISP). A total of 1809 SARI hospitalizations (29.4%) were laboratory confirmed with A/H1N1 pandemic influenza, giving a positivity rate (A/H1N1-SARI/tested SARI) of 53.6%. For each hospitalized patient, we obtained age, gender, reporting region, and date of symptoms onset.

### Population data

Previous studies have found population size to be partly correlated with spatial variation in timing of the 2009 A/H1N1 pandemic across geographic units (e.g. [1,2,22]). We obtained regional estimates of population size for 2009 from the Instituto Nacional de Estadísticas [23].

### Climate data

**Figure 1:**
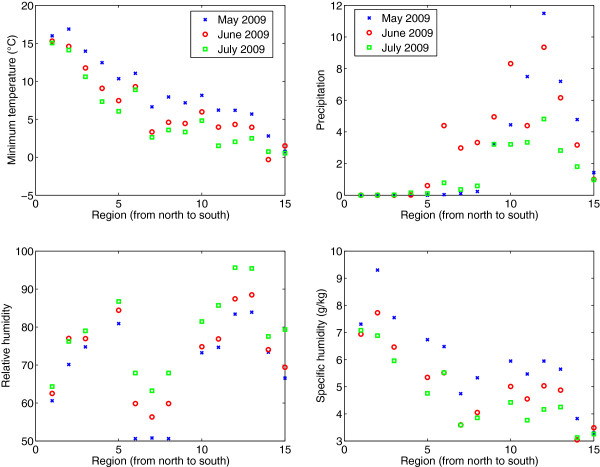
Average minimum temperature, maximum temperature, precipitation, and specific humidity for the months of May, June, and July 2009 across Chilean regions as reported by the Dirección Meteorológica de Chile.

Transmission of influenza in the laboratory setting has been found to be significantly associated with temperature and humidity levels [24-29]. To evaluate the link between pandemic influenza transmission and meteorological conditions in Chile, we collected daily regional time series of minimum and maximum temperature, precipitation, relative humidity, and specific humidity, reported by local meteorological stations for 2009 to the Dirección Meteorológica de Chile (Figure 1) [30]. Because relative humidity and specific humidity data were not available for all 15 regions, we supplemented our climate data using information from the National Oceanic and Atmospheric Administration records, accessible from the Weather Underground website ( http://www.wunderground.com/). As a reference, in May 2009 during the initial pandemic growth phase in Chile, the minimum (respectively, maximum) temperature ranged from 0.77°C (6.4°C) in the southernmost region of Magallanes to 16°C (22°C) in the northernmost region of Arica y Parinacota. Average precipitation was almost null in the 5 northernmost regions and highest in the Southern region of Los Rios (11.5 mm). Specific humidity varied from 3.3 g/kg in the southernmost region of Magallanes to 9.3 g/kg in the northern region of Tarapacá (Additional file 1: Figure S1). 

### International flight passenger data

We obtained data on the monthly number of international passengers arriving to international airports in Chile in 2008 and 2009 from Junta de Aeronáutica Civil [31]. We used 2008 data as a control for the potential impact of the pandemic on air travel. A total of 166,832 international passengers arrived to international airports of Chile in April 2009, representing a 1.5% reduction relative to April 2008. A larger reduction in incoming international air traffic was observed in May 2009 relative to May 2008 (12.5%).

### International arrival data at regional seaports

We obtained data on the monthly number of international arrivals at Chilean seaports in 2009 from the Dirección General de Terrirorio Marítimo y Marina Mercante de la Fuerza Armada de Chile [32]. A total of 581 and 552 international arrivals were documented at Chilean seaports in April and May 2009, respectively.

### Statistical analysis

#### Geographic patterns

**Figure 2:**
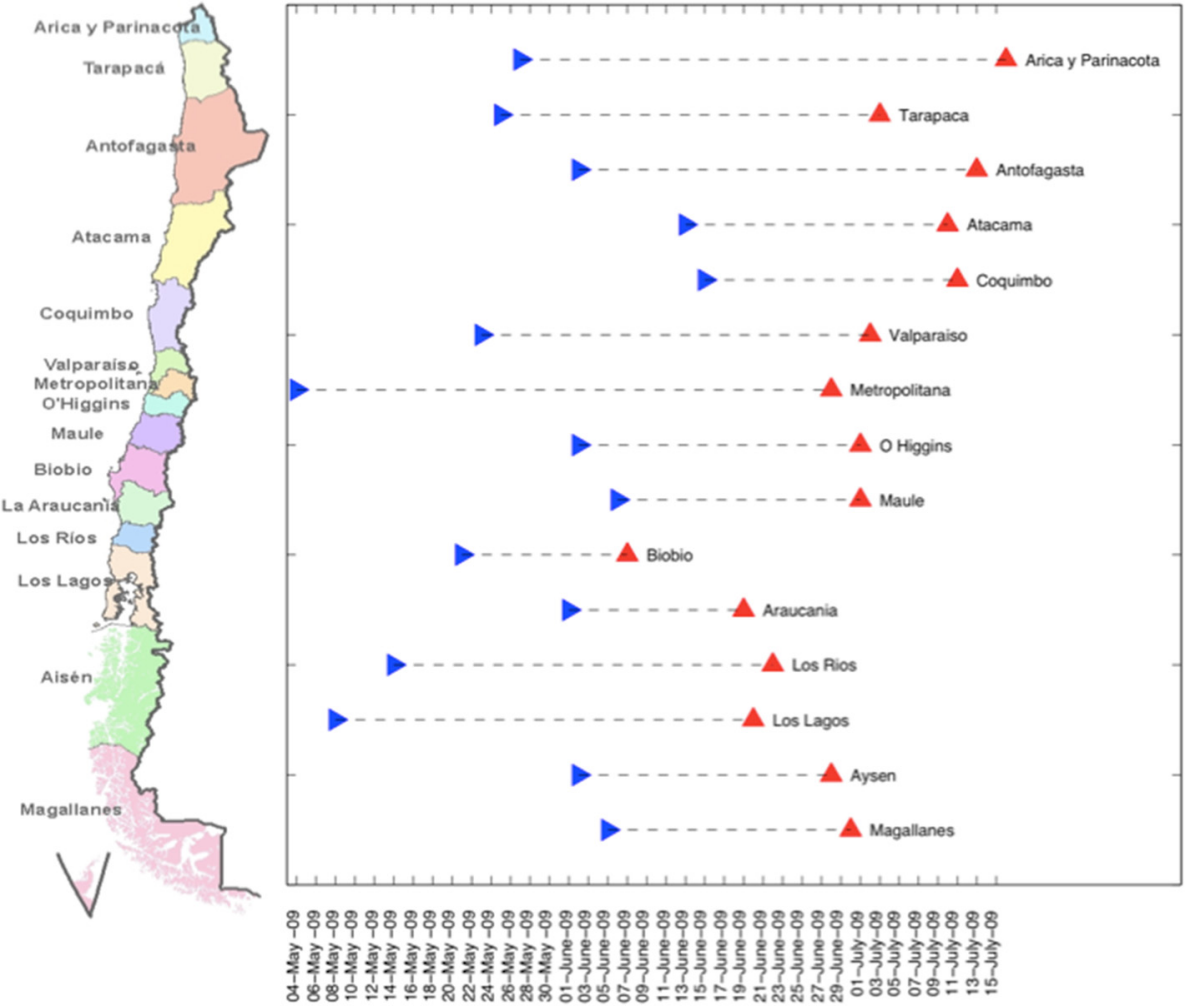
Pandemic onset (denoted by symbol >) and pandemic peak (denoted by symbol ^) timing across the 15 Chilean regions sorted from north (top) to south (bottom) Chile.

We analyzed region- and age-specific time series of A/H1N1-positive SARI hospitalizations by day of symptom onset to analyze the geographic dissemination patterns of the pandemic across Chile (Figure 2). For each region, we recorded the cumulative number of A/H1N1-positive SARI hospitalizations during Apr-Dec 2009, the date of pandemic onset defined by the first A/H1N1-specific SARI hospitalization, and the date of pandemic peak defined as the date with maximal incidence by geographic regions. All regions experienced a single peak of pandemic activity. We analyzed the association between the dates of pandemic onset and peak timing with population size, latitude of population centres, climatic factors averaged during the exponential growth phase by geographic region and international inflow of air traffic and arrivals at seaports in May 2009 using univariate Spearman correlations. Finally, we built a multivariate linear regression model with all predictor variables to disentangle the factors explaining geographical variation in pandemic onset and peak timing across Chilean regions. We generated a simplified model by means of a backward stepwise elimination procedure. As a sensitivity analysis, we repeated these analyses with all SARI hospitalizations.

#### Spatial autocorrelation

We also quantified the extent of spatial autocorrelation across geographic regions in our epidemiological data using Moran’s I statistic [33] which is given by: $I=\frac{n\sum_{i=1}^{n}\sum_{j=1}^{n}w_{\mathrm{ij}}z_{i}z_{j}}{S_{0}\sum_{i=1}^{n}z_{i}^{2}}\;\text{(i}\neq \text{j})$ where *n=15* is the number of Chilean regions, and *w*_*ij*_ is a nearest-neighbor spatial matrix where *w*_*ij*_*= 1* whenever regions i and j share borders (contiguous regions) and equals 0 otherwise. In addition, $z_{i}=x_{i}-\bar{x}$ where *x*_*i*_ is the number of A/H1N1-positive SARI hospitalizations per 100,000 people in region i, $\bar{x}$ is the mean incidence across regions and *S*_*0*_ is a normalization constant, such that $S_{0}=\sum_{i=1}^{n}\sum_{j=1}^{n}w_{\mathrm{ij}}\;\text{(i}\neq \text{j})$. We explored spatial autocorrelation during the main pandemic months of May, June and July 2009. We assessed statistical significance via randomization by generating an empirical null distribution (no-auto-correlation) given by generating 10,000 artificial time series by permuting regions of the original data. That is, statistical significance was evaluated under the assumption that the statistics computed using the observed data was sampled from the reference distribution [34].

#### Impact of winter vacation period

**Figure 3:**
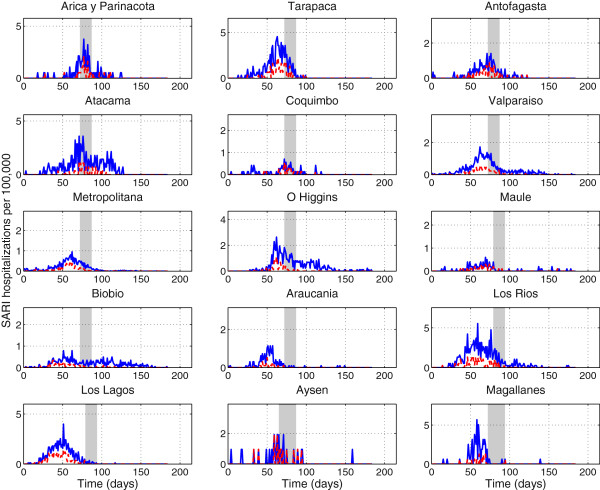
**Daily number of SARI hospitalizations (solid blue line) and laboratory-confirmed A/H1N1 influenza SARI hospitalizations (dashed red line) per 100,000 people for 15 regions of Chile sorted from north to south Chile, May-October 2009.** The grey shaded area indicates the region-specific winter school vacation period.

School activities have been found to be significantly correlated with influenza transmission rates [3,35-37] and changes in age distribution of pandemic influenza incidence patterns [1,2,38]. We evaluated the geographic specific effect of the school closing period by exploring daily trends in the ratio of incident A/H1N1-positive SARI hospitalizations among the student population (5–20 years) to incident cases among all other age groups. The winter vacation period started on July 11, 2009 in most regions of Chile and coincided with the peak timing in the northern regions as shown in Figure 3. To avoid low case counts we aggregated the 15 region-specific time series into 3 broad geographic areas referred to as northern, central and southern areas. 

#### Estimation of the reproduction number

Because region-specific time series were generally sparse during the initial pandemic phase, we estimated the reproduction number of the 2009 A/H1N1 influenza pandemic across Chile using daily time series of A/H1N1-positive SARI hospitalizations for the 3 broad geographic areas (northern, central and south Chile). In the early stages of an epidemic, the epidemic grows exponentially, as the effect of increasing incidence on the depletion of the susceptible population remains small [39-41]. The exponential growth rate, r, can be estimated from the exponential growth phase of the pandemic, using a Poisson maximum likelihood method as explained in the Additional file 2: Supplement. The reproduction number can be derived by substituting the estimate for “r” into an expression derived from the linearization of the classical Susceptible-Exposed-Infectious-Recovered (SEIR) transmission model [41,42]:

(1) $$\text{R=(1+}\frac{\text{r}}{{\text{b}}_{1}})(1+\frac{\text{r}}{{\text{b}}_{2}})$$

where 1/b_1_ and 1/b_2_ are respectively the mean latent and infectious periods which are assumed to be exponentially distributed. Hence, the mean generation interval between two successive cases is given by *T*_*c*_ = 1/*b*_1_ + 1/*b*_2_. We assumed a mean generation interval of three (1/b_1_ = 1.5 days and 1/b_2_ = 1.5 days) and four days (1/b_1_ = 2 days and 1/b_2_ = 2 days) [43-46]. As a sensitivity analysis, we also obtained an upper bound R estimate for the extreme case of a fixed generation interval [41].

This study is covered by a formal collaborative agreement signed between the first author and representatives of the Chilean Ministry of Health. This study did not require ethics committee approval; epidemiological data were de-identified. Data employed in this study were collected for epidemiological surveillance purposes.

## Results

### Overall description of pandemic activity, May-Dec 2009

The first case of 2009 A/H1N1 influenza infection in Chile was reported on May 17th, 2009, coinciding with the fall season (see Additional file 1: Table S1 for a chronology of main events). Of note, the Chilean Ministry of Health recommended against non-essential travel to the US and Mexico after the initial pandemic alert on May 17th, and epidemiological surveillance was reinforced.

The time series of daily number of SARI hospitalizations and A/H1N1-positive SARI hospitalizations are shown in Figures 2 and 3 for the 15 regions of Chile . The incidence curves of SARI hospitalizations were significantly correlated with those of A/H1N1-positive SARI hospitalizations (Figure 3, Spearman rho = 0.86, P < 0.0001. The pandemic A/H1N1 virus spread asynchronously across the 15 Chilean regions, following a South to North travelling pattern (Figure 2). The exponential growth phase consisted of 38 days for the northern geographic area (May 18th to June 24th), 30 days for the central area (May 18th to June 16th) and 18 days for the southern area (May 18th to June 4th).

We did not detect significant spatial autocorrelation in our region-specific time series of A/H1N1-positive SARI hospitalizations during the highest incidence months of May (P = 0.61), June (P = 0.09), or July (P = 0.60). We obtained similar results when using the time series of all SARI hospitalizations (P > 0.12).

### Timing of pandemic onset

The onset date of the first SARI hospitalization across Chilean regions was associated with population size, with more populous regions experiencing earlier pandemic onset than low population regions (Spearman rho = −0.61, P = 0.02), but this correlation did not reach statistical significance using A/H1N1-positive SARI hospitalizations (Spearman rho = −0.4, P = 0.14). Moreover, pandemic onset was not correlated with latitude (rho = −0.14, P = 0.61), inflow of international air traffic passengers (rho = 0.3, P = 0.28) or the number of international arrivals at Chilean seaports (rho = 0.18, P = 0.52) in the first month of the pandemic in May 2009. The timing of local pandemic onset was not significantly correlated with climatological variables including minimum temperature (rho = 0.15, P = 0.62), maximum temperature (rho=−0.001, P=0.99), precipitation (rho = −0.3, P = 0.30), relative humidity (rho = −0.05, P = 0.89) and specific humidity (rho = −0.08, P = 0.80), after adjusting for population size. Our sensitivity analysis based on all SARI hospitalizations gave similar results to those obtained using A/H1N1-positive SARI hospitalizations.

### Timing of pandemic peak

We found a strong latitudinal gradient in pandemic peak timing identified from daily A/H1N1-positive SARI hospitalizations, with Southern regions experiencing earlier pandemic activity than Northern regions (Spearman rho = 0.80, P = 0.0002; Figure 2). A similar pattern was observed in the time series of all SARI hospitalizations (rho = 0.83, P = 0.0001). Specifically, the southernmost regions (Biobio, Araucania, Los Rios, Los Lagos, Aysen, and Magallanes) peaked 16-39-days earlier relative to the northernmost region (Arica y Parinacota). A similar geographical pattern was found in weekly reports of influenza-like-illnesses across 29 influenza sentinel units covering northern, central and southern regions of Chile (correlation between peak influenza-like-illness and latitude, Spearman rho = 0.43, P = 0.02; Additional file 1: Figures S2–S3). Next, we assessed the role of climate conditions, demographic factors, and international connectivity patterns in driving geographical variations in pandemic peak timing.

Peak timing was significantly correlated with most climatic factors studied during the exponential growth phase by geographic region including minimum temperature (Spearman rho = 0.64, P = 0.009), maximum temperature (Spearman rho = 0.59, P = 0.02), precipitation (Spearman rho = −0.88, P < 0.0001), and relative humidity (Spearman rho = −0.70, P = 0.008). In contrast, pandemic peak timing was not correlated with specific humidity (Spearman rho = 0.34, P = 0.25), population size (Spearman rho = −0.37, P = 0.18), incoming international air travel passengers (Spearman rho=0.08, P = 0.77), or incoming international seaport arrivals (Spearman rho = −0.005, P = 0.98). Stepwise multivariate regression identified maximum temperature, specific humidity and latitude as the only significant predictors of regional pandemic peak timing (R^2^ = 68.5%, P = 0.01, Table 1), with earlier peak timing occurring in colder regions and at lower levels of specific humidity. Our sensitivity analysis based on the time series of all SARI hospitalizations gave consistent results; using all SARI hospitalizations the best model with maximum temperature, specific humidity and latitude explained 79.7% of the variability in peak timing (P = 0.002, Table 2).

**Table 1:** Best-fit multivariate linear regression model of peak timing in A/H1N1-positive SARI hospitalizations derived via backward elimination procedure

**Predictor variable**	**Coefficient (95% CI)**	**Coefficient of variation (R**^**2**^**)**	**P value**
**Maximum temperature**	−2.80 (−5.1, -0.47)	68.5%	0.01
**Specific humidity**	−5.96 (−11.27, -0.65)		
**Latitude**	2.47 (1.08, 3.87)		
**Intercept**	221.41 (121.56, 321.26)		

**Table 2:** Best-fit multivariate linear regression model of peak timing in all SARI hospitalizations derived via backward elimination procedure

**Predictor variable**	**Coefficient (95% CI)**	**Coefficient of variation (R**^**2**^**)**	**P value**
**Maximum temperature**	−1.93 (−3.34, -0.51)	79.7%	0.002
**Specific humidity**	−4.5 (−7.75, -1.26)		
**Latitude**	1.9 (1.05, 2.76)		
**Intercept**	181.68 (120.61, 242.75)		

### Reproduction number

**Figure 4:**
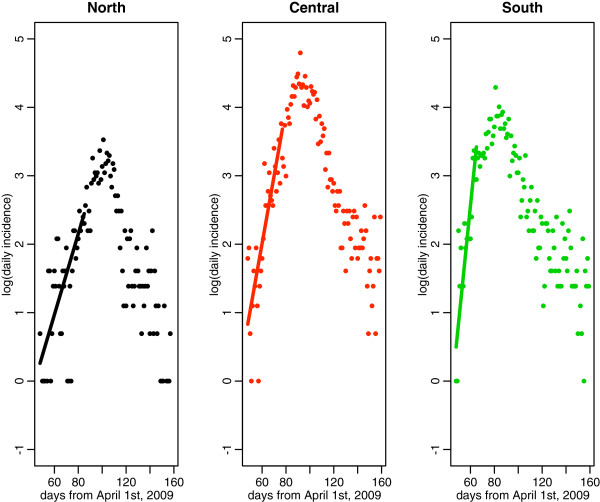
**Exponential model fits to the incidence data (in logarithmic scale) across northern, central and southern areas of Chile.** Data are the dots and the lines indicate the best fit of the exponential model to the exponential rise portion of the incidence curves as described in the supplementary document.

We estimated the mean R for the northern, central and southern geographic areas based on daily laboratory-confirmed A/H1N1-positive SARI hospitalizations. The exponential growth phase consisted of 38 days for the northern area (May 18th to June 24th), 30 days for the central region (May 18th to June 16th) and 18 days for the southern region (May 18th to June 4th (Figure 4). The corresponding R estimates for the northern, central, and southern geographic areas were 1.19 (95% CI: 1.13, 1.24), 1.32 (1.27, 1.37), and 1.58 (1.45, 1.72), respectively assuming a three day mean generation interval, and 1.25 (95% CI: 1.18, 1.32), 1.43 (1.36,1.50), and 1.81 (1.62, 2.0), respectively assuming a four day mean generation interval. The results show a decreasing gradient in R from southern to northern Chile. We perform pair-wise Pearson-χ^2^ comparisons of the R estimates to test the null hypothesis that the estimates are drawn from the same mean. The pair-wise comparisons revealed significant geographical differences between regions at P < 0.001 level or better, with the largest difference being between the southern and northern regions at P < 0.0001. Upper bound R estimates based on a fixed generation interval yielded slightly higher values (Table 3).

**Table 3:** Mean estimates of the reproduction number and corresponding 95% confidence intervals for the 2009 A/H1N1 influenza pandemic by geographic region

**Distribution of generation interval**	**Geographic area**					
	**Northern area**		**Central area**		**Southern area**	
	**3-day serial interval**	**4-day serial interval**	**3-day serial interval**	**4-day serial interval**	**3-day serial interval**	**4-day serial interval**
**Exponentially-distributed**	1.19 (1.13, 1.24)	1.25 (1.18, 1.32)	1.32 (1.27, 1.37)	1.43 (1.36, 1.50)	1.58 (1.45, 1.72)	1.81 (1.62, 2.0)
**Fixed generation interval**	1.19 (1.14, 1.25)	1.27 (1.19, 1.35)	1.34 (1.29, 1.40)	1.48 (1.40, 1.57)	1.68 (1.50, 1.87)	1.99 (1.72, 2.30)

The epidemic growth phase used to estimate the reproduction number consisted of 38 days for the northern area (May 18th to June 24th), 30 days for the central area (May 18th to June 16th) and 18 days for the southern area (May 18th to June 4th).

### Impact of winter school vacation period

**Figure 5:**
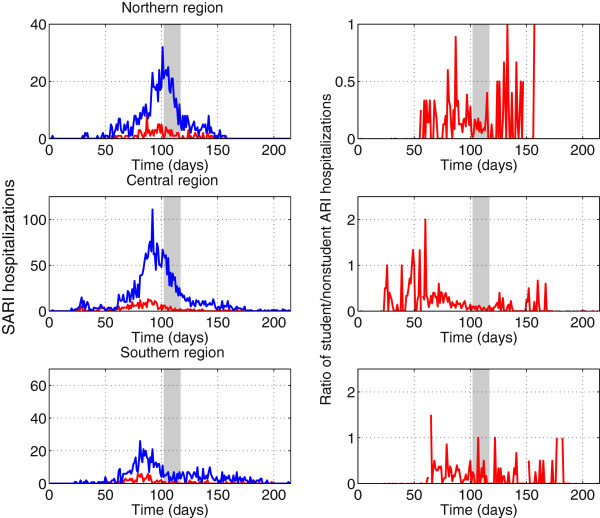
**Changes in the age distribution of SARI hospitalizations in Chile, May-December 2009.** Daily time series of SARI hospitalizations among students (5–20 years, red curve) and other age groups (blue curve) and daily ratio of student to nonstudent SARI hospitalizations. The grey shaded area indicates the winter school vacation period from July 11th to July 26th, 2009.

School vacation were synchronous across Chile, started on July 11, 2009, and lasted for 15 days. To assess the potential impact of school vacation on pandemic transmission, we evaluated trends of A/H1N1-positive SARI hospitalizations among students and non-student popualtions. At the national scale, the ratio of student to non-student cases decreased significantly by 76% during the 15-day winter vacation period relative to the preceding 15-day period (Wilcoxon test, P = 0.001). Given the asynchrony in pandemic activity across Chile, the school vacation period coincided with different phases of the pandemic in different regions. In the northern area, the pandemic peaked at about the same time as the start of the winter vacation period. In contrast, pandemic activity in the central and southern regions had substantially declined when the winter vacation period began (Figure 5). Accordingly, the change in age distribution of cases associated with the winter vacation period was most pronounced in the North (220% reduction in the student to non-student ratio; Wilcoxon test, P = 0.02), intermediate in the central geographic area (75% reduction, Wilcoxon test, P = 0.03), and non-existent in the Southern area (Wilcoxon test, P = 0.2).

## Discussion

To the best of our knowledge this is the first study to explore the relationship between spatio-temporal variation in the dynamics of the 2009 A/H1N1 pandemic and demographic and climatic factors, and international travel patterns across a large range of latitudes. We found that the 2009 A/H1N1 pandemic influenza in Chile was characterized by a South to North gradient of increasingly late peak activity and decreasing disease transmissibility. Geographical variation in pandemic peak timing was associated with differences in latitude and climatic conditions, with latitude, maximum temperature and specific humidity accounting for 69-80% of the variability. Our findings could have important public health implications. In particular, intensified surveillance strategies in southern regions could lead to earlier detection of novel influenza viruses and improved pandemic control outcomes.

The latitudinal gradient evidenced in laboratory–confirmed A/H1N1 SARI hospitalizations in this study is confirmed by geographical patterns in weekly incidence of influenza-like-illnesses reported to the Chilean Ministry of Health (Additional file 1: Figures S2–S3). In addition, our data are consistent with previous studies of laboratory-confirmed A/H1N1 influenza reporting peak pandemic activity on June 22, 2009 in the central region of Santiago [47], and on June 7–13, 2009, in the southern city of Puerto Montt [48].

The south–north gradient in peak timing observed in our study is consistent with a decreasing trend in the reproduction number in the same direction and is statistically associated with maximum temperature and specific humidity. These findings are in agreement with experimental studies suggesting that influenza transmission is more efficient under dry and cold conditions [24-29]. In particular, Chile experienced a south–north gradient in climatic conditions during May 2009 as pandemic activity was building up, ranging from 3.3 to 9.3 g/kg for specific humidity and 6.4°C to 22°C for maximum temperature.

The northward gradient of 2009 pandemic activity evidenced in Chile is reminiscent of the spread of the 2009 pandemic in Brazil, with the Southernmost regions of this country being hit earlier and experiencing greater severity than the Northernmost regions [49]. In contrast, seasonal influenza originates from low-population regions in the equatorial North of Brazil and travels to highly populous regions in the subtropical South over a 3-month period [50], together with a weak transmissibility gradient [51]. In light of the intriguing Brazilian experience, it would be interesting to contrast the spread of the 2009 pandemic with that of seasonal influenza in Chile. Unfortunately, SARI surveillance data was limited to the pandemic period in Chile and no comparable information exists for prior years.

Although the main period of pandemic activity in Chile seems primarily correlated with local climatic conditions, we note that the timing of introduction of the first A/H1N1 cases (pandemic ‘onset’) was weakly associated with population size, with larger population centers experiencing earlier introductions than less populous regions. This hierarchical pattern of spread is in agreement with seasonal influenza epidemics in the United States [52], the 2009 A/H1N1 influenza pandemic in Peru [2] and Mexico [1], and the 1918 influenza pandemic in England and Wales [53,54]. The Chilean experience also suggests that despite early introduction of the A/H1N1 virus in a large population center like Santiago in May 2009, located in the center of the country, local climatic conditions were not favorable for immediate full-scale transmission of the pandemic virus.

Winter school vacation period started on July 11th and coincided with the beginning of the downward phase of the pandemic in northern regions, similar to the 2009 pandemic experience in Peru [2]. In contrast, pandemic activity was well into the declining phase by the time winter vacations started in southern and central regions. Accordingly, the shift in the age distribution of cases associated with the winter vacation period was most pronounced in the northern area. A similar pattern has been reported in previous studies [1,2]. Of note, a widespread teachers strike involving public schools across Chile (approximately from May 18th to June 8th, 2009) could have slowed down the initial growth rate of the pandemic, although we were not able to quantify the effect of the strike here.

Our reproduction number estimates are consistent with previous studies reporting estimates in the range 1.2-2.1 for Chile, using national ILI and laboratory-confirmed influenza A/H1N1 cases [5,20,55]. Further, R was estimated to be 1.8 (95% CI: 1.6, 2.0) for the southern region of Puerto Montt [48], in line with our R estimate for Chile’s southern region (R~ 1.6-2.0). Overall, our transmissibility estimates for Chile are consistent with the range of global estimates reported for community-based settings at 1.2-2.4 [1,2,5,18,43,48,56-63], while higher estimates have been obtained in school settings [2,44,64,65]. The surprising gradient of pandemic A/H1N1 transmissibility observed from South to North Chile could be associated with differences in climatic conditions, prior immunity, or baseline differences in influenza transmission between regions, similarly to those reported for Brazil [51]. Unfortunately, the resolution of our data did not allow for more detailed analysis.

Chile experienced a single pandemic wave in 2009 as did other Southern Hemisphere countries including Argentina [11], Australia [12,13], New Zealand [12] and Peru [2]. Other countries experienced multiple pandemic waves, including Mexico, the United States, and the United Kingdom [9,10,66]. The timing of pandemic waves has been correlated with population density, school cycles and the season in which the novel influenza virus is introduced into local populations [1,2]. Our 2009 pandemic study set in Chile adds to our current understanding of the role of climatic conditions in modulating the transmission dynamics of pandemic influenza.

Several strengths and caveats of our study are worth noting. We used data on all SARI hospitalizations as well as a subset of hospitalizations that were laboratory-confirmed for A/H1N1 infections, representing all public and private hospitals in Chile. Testing rates for A/H1N1 influenza remained at 35% throughout the pandemic and were comparable to those of other countries [1]. Moreover, national respiratory virus surveillance for influenza-like-illness demonstrated that A/H1N1 predominated among other respiratory viruses in 96% of individuals aged 5 years and over [47]. In contrast, RSV co-circulated with A/H1N1 influenza among children < 5 years, although influenza remained the dominant virus in this age group [18,47]. Because a total of 365 (5.7%) of the SARI hospital records were missing the date of symptoms onset, we used the date of notification for 197 of these records for which the date of notification was available. Differences in reporting across regions cannot be ruled out, although there was no evidence of weaker reporting rates in less populous regions. On the contrary, regions with lower population sizes reported more SARI hospitalizations proportionally than larger regions in Chile. We note that our estimates of the reproduction number were derived using simple methodology relying on the initial growth rate of the pandemic across geographic areas of Chile [41,67]. More detailed epidemiological data providing information on the number of imported cases could have allowed the use of more refined estimation methods, but were not available to us (see e.g., [68,69]).

## Conclusions

In conclusion, there was a lag in the period of most intense 2009 pandemic influenza activity from southern to northern regions of Chile, significantly associated with geographical differences in maximum temperature and specific humidity. The latitudinal gradient in timing of pandemic activity was accompanied by a gradient in reproduction number (P < 0.0001). These two findings suggest that meteorological conditions may have modulated the transmissibility of 2009 A/H1N1 influenza in Chile. Our results could have significant public health implications for pandemic influenza control. Specifically, intensified surveillance strategies in southern regions could lead to early detection of pandemic influenza viruses and improved control outcomes. Furthermore, the spatial differences in the timing of local pandemic influenza in our study demonstrate the advantages of using high-resolution data to detect heterogeneous pandemic patterns. More studies are needed to determine whether these findings may be generalized to seasonal influenza epidemics and may be integrated in pandemic preparedness scenarios.

## Competing interests

Authors declare no competing interests relevant to this study.

## Authors’ contributions

GC, ST and CV designed the study. RF, VS, CV, MC, JV, and AO participated in data acquisition. GC and ST analyzed the data and wrote the first draft of the manuscript. GC, ST, CV, RF, VS, LS, MM, and ML participated in the interpretation of results. All authors contributed to the writing and editing of the manuscript.

## Pre-publication history

The pre-publication history for this paper can be accessed here:

http://www.biomedcentral.com/1471-2334/12/298/prepub

## Supplementary Material

Additional file 1 **Table S1.** Timeline of events relevant to the detection, control, and school activities during the 2009 influenza pandemic in Chile. **Figure S1**. Daily average minimum temperature in northern, central and southern regions of Chile. The northern geographic area comprises the 5 northernmost regions of: 1) Arica y Parinacota, 2) Tarapacá, 3) Antofagasta, 4) Atacama, and 5) Coquimbo; the broad central area includes the regions of 1) Valparaíso, 2) Metropolitana, 3) O’Higgins, and 4) Maule; and the broad southern geographic area includes the southernmost regions of 1) Bíobío, 2) Araucanía, 3) Los Ríos, 4) Los Lagos, 5) Aysén, and 6) Magallanes. **Figure S2.** Pandemic peak timing based on weekly time series of influenza-like-illness (ILI) cases for 29 Chilean ILI sentinel sites of Chile during 52 epidemiological weeks in 2009 as reported to the Chilean Ministry of Health and shown in geographic order from north (top) to south (bottom). We found a significant shift in the peak timing from southern to northern provinces (Spearman rho = 0.43, P = 0.02). Provinces from north to south: Arica, Iquique, Antofagasta, Atacama, Coquimbo, Valparaíso - San Antonio, Viña del Mar - Quillota, Aconcagua, Metropolitano Norte, Metropolitano Occidente, Metropolitano Central, Metropolitano Oriente, Metropolitano Sur, Metropolitano Sur Oriente, O'Higgins, Maule, Ñuble, Concepción, Talcahuano, Bio Bio, Arauco, Araucanía Norte, Araucanía Sur, Valdivia, Osorno, Del Reloncaví, Aysén, Magallanes, Chiloé. **Figure S3.** Weekly number of consolidated influenza-like-illness (ILI) cases in northern, central and southern geographic regions of Chile in 2009. The northern region is comprised by provinces: Arica, Iquique, Antofagasta, Atacama, Coquimbo; central region provinces: Valparaíso - San Antonio, Viña del Mar - Quillota, Aconcagua, Metropolitano Norte, Metropolitano Occidente, Metropolitano Central, Metropolitano Oriente, Metropolitano Sur, Metropolitano Sur Oriente, O'Higgins, Maule; southern region provinces: Ñuble, Concepción, Talcahuano, Bio Bio, Arauco, Araucanía Norte, Araucanía Sur, Valdivia, Osorno, Del Reloncaví, Aysén, Magallanes, Chiloé. Click here for file

Additional file 2 Estimation of the reproduction number. Click here for file
