# Supplementary material for: The influence of climatic conditions on the transmission dynamics of the 2009 A/H1N1 influenza pandemic in Chile
Source: BMC Infect Dis. 2012 Nov 13;12:298. doi: 10.1186/1471-2334-12-298 (PMC3518181; doi:10.1186/1471-2334-12-298)

**Supplementary Tables**

**Table S1. Timeline of events relevant to the detection, control, and school activities during the 2009 influenza pandemic in Chile.**

| **Dates** | **Events** |
| --- | --- |
| April 23, 2009 | Chilean government issues educational campaigns to increase awareness among the population on the role of personal hygiene measures for prevention and ordered fever screening for all travelers from Mexico and the United States at international airports. |
| April 29, 2009 | The World Health Organization raises the pandemic alert to level 5 after sustained dissemination of the pandemic virus in several countries. |
| May 17, 2009 | First confirmed case with pandemic A/H1N1 influenza in a person returning from Dominican Republic. |
| May 18-June 8, 2009 | Public schools were closed throughout Chile due to a general teacher’s strike. |
| June 1, 2009 | Sustained community transmission is observed; public health authorities shift from a containment to a mitigation phase. |
| July 11, 2009 | Winter school break starts in most regions of the country (see Figure 3) |

**Supplementary Figures**

**Supplementary Figure 1** Daily average minimum temperature in northern, central and southern regions of Chile. The northern geographic area comprises the 5 northernmost regions of: 1) Arica y Parinacota, 2) Tarapacá, 3) Antofagasta, 4) Atacama, and 5) Coquimbo; the broad central area includes the regions of 1) Valparaíso, 2) Metropolitana, 3) O’Higgins, and 4) Maule; and the broad southern geographic area includes the southernmost regions of 1) Bíobío, 2) Araucanía, 3) Los Ríos, 4) Los Lagos, 5) Aysén, and 6) Magallanes.


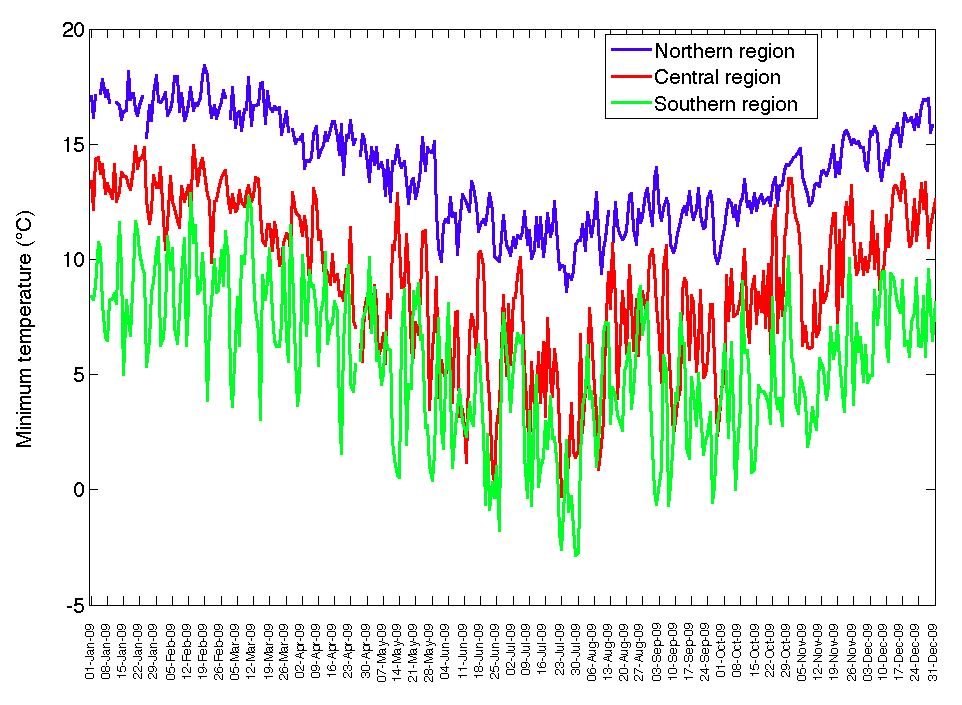


Daily average maximum temperature in northern, central and southern regions of Chile.


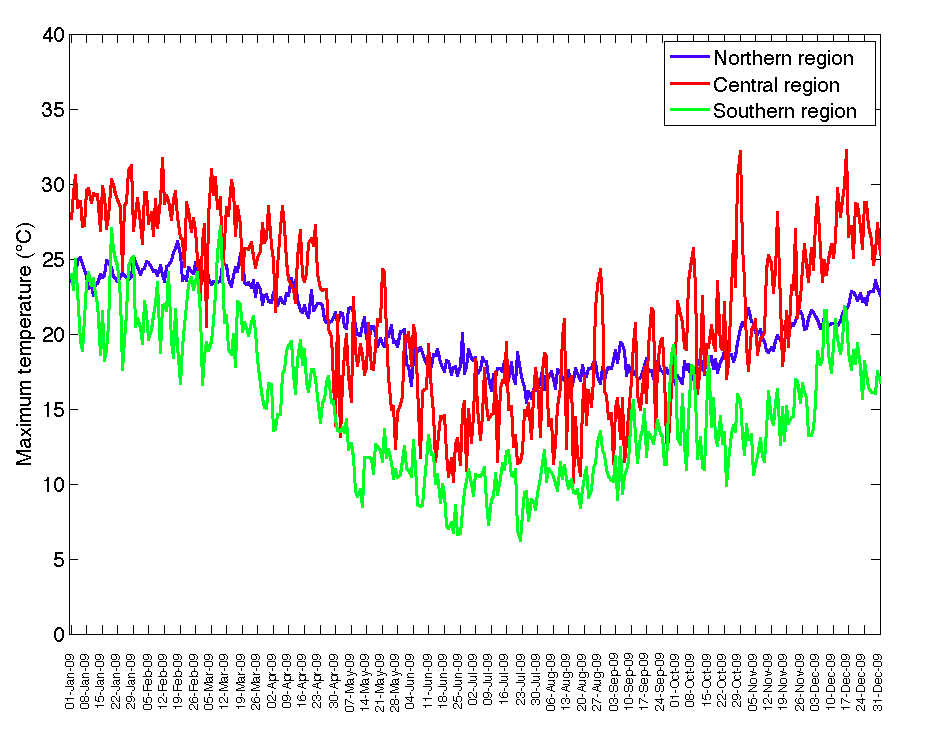


Daily average precipitation in northern, central and southern regions of Chile.


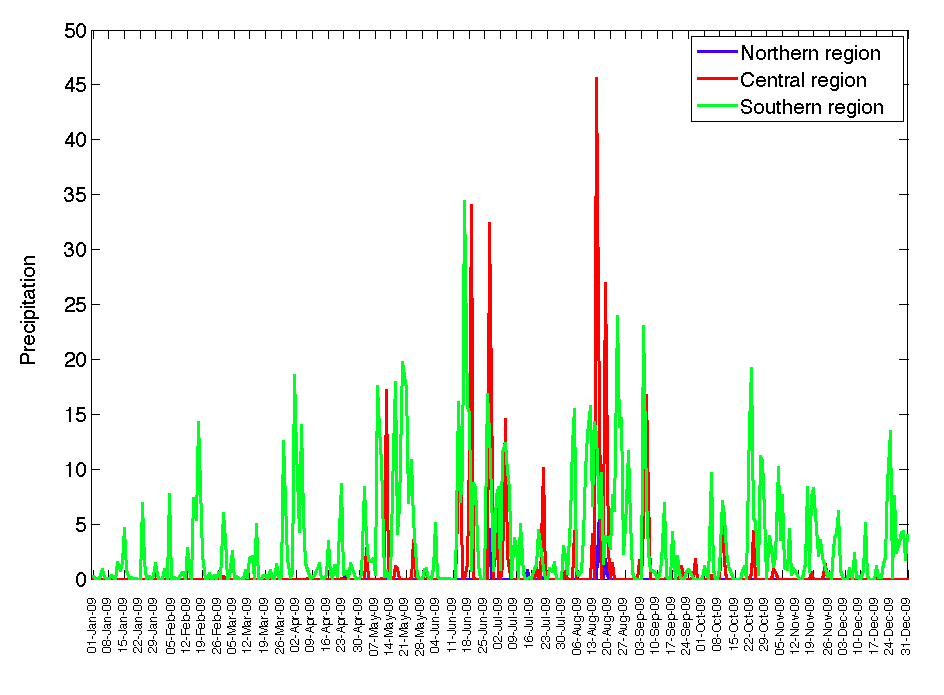


Daily average relative humidity in northern, central and southern regions of Chile.


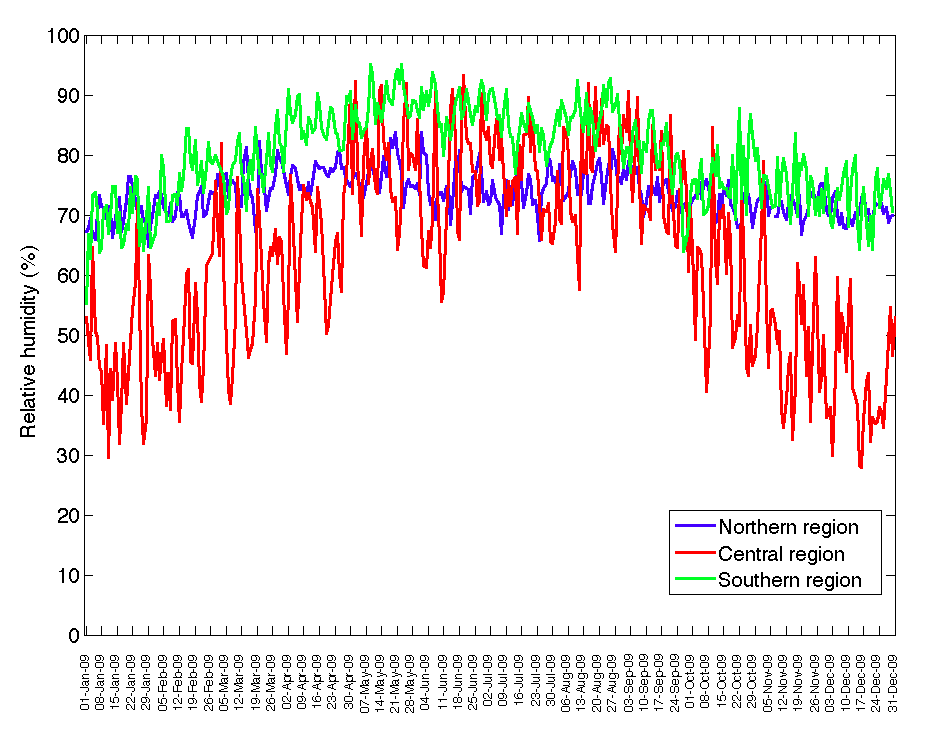


Daily average specific humidity in northern, central and southern regions of Chile.


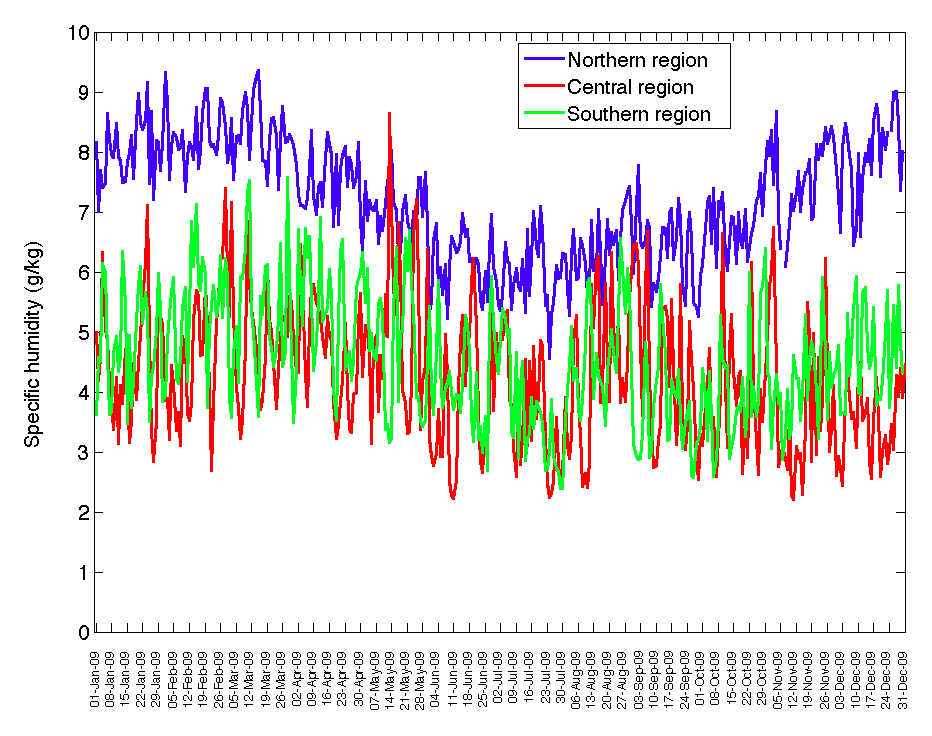


**Supplementary Figure 2** Pandemic peak timing based on weekly time series of influenza-like-illness (ILI) cases for 29 Chilean ILI sentinel sites of Chile during 52 epidemiological weeks in 2009 as reported to the Chilean Ministry of Health and shown in geographic order from north (top) to south (bottom). We found a significant shift in the peak timing from southern to northern provinces (Spearman rho=0.43, P=0.02).

Provinces from north to south: Arica, Iquique, Antofagasta, Atacama, Coquimbo, Valparaíso - San Antonio, Viña del Mar - Quillota, Aconcagua, Metropolitano Norte, Metropolitano Occidente, Metropolitano Central, Metropolitano Oriente, Metropolitano Sur, Metropolitano Sur Oriente, O'Higgins, Maule, Ñuble, Concepción, Talcahuano, Bio Bio, Arauco, Araucanía Norte, Araucanía Sur, Valdivia, Osorno, Del Reloncaví, Aysén, Magallanes, Chiloé

**Supplementary Figure 3** Weekly number of consolidated influenza-like-illness (ILI) cases in northern, central and southern geographic regions of Chile in 2009. The northern region is comprised by provinces: Arica, Iquique, Antofagasta, Atacama, Coquimbo; central region provinces: Valparaíso - San Antonio, Viña del Mar - Quillota, Aconcagua, Metropolitano Norte, Metropolitano Occidente, Metropolitano Central, Metropolitano Oriente, Metropolitano Sur, Metropolitano Sur Oriente, O'Higgins, Maule; southern region provinces: Ñuble, Concepción, Talcahuano, Bio Bio, Arauco, Araucanía Norte, Araucanía Sur, Valdivia, Osorno, Del Reloncaví, Aysén, Magallanes, Chiloé.


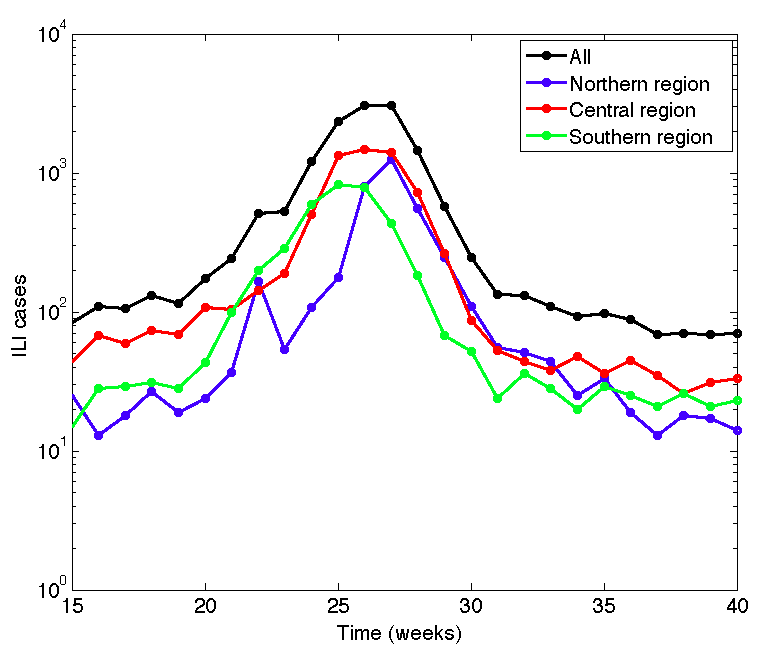

Supplement: Additional file 1 — Table S1. Timeline of events relevant to the detection, control, and school activities during the 2009 influenza pandemic in Chile. Figure S1. Daily average minimum temperature in northern, central and southern regions of Chile. The northern geographic area comprises the 5 northernmost regions of: 1) Arica y Parinacota, 2) Tarapacá, 3) Antofagasta, 4) Atacama, and 5) Coquimbo; the broad central area includes the regions of 1) Valparaíso, 2) Metropolitana, 3) O’Higgins, and 4) Maule; and the broad southern geographic area includes the southernmost regions of 1) Bíobío, 2) Araucanía, 3) Los Ríos, 4) Los Lagos, 5) Aysén, and 6) Magallanes. Figure S2. Pandemic peak timing based on weekly time series of influenza-like-illness (ILI) cases for 29 Chilean ILI sentinel sites of Chile during 52 epidemiological weeks in 2009 as reported to the Chilean Ministry of Health and shown in geographic order from north (top) to south (bottom). We found a significant shift in the peak timing from southern to northern provinces (Spearman rho = 0.43, P = 0.02). Provinces from north to south: Arica, Iquique, Antofagasta, Atacama, Coquimbo, Valparaíso - San Antonio, Viña del Mar - Quillota, Aconcagua, Metropolitano Norte, Metropolitano Occidente, Metropolitano Central, Metropolitano Oriente, Metropolitano Sur, Metropolitano Sur Oriente, O'Higgins, Maule, Ñuble, Concepción, Talcahuano, Bio Bio, Arauco, Araucanía Norte, Araucanía Sur, Valdivia, Osorno, Del Reloncaví, Aysén, Magallanes, Chiloé. Figure S3. Weekly number of consolidated influenza-like-illness (ILI) cases in northern, central and southern geographic regions of Chile in 2009. The northern region is comprised by provinces: Arica, Iquique, Antofagasta, Atacama, Coquimbo; central region provinces: Valparaíso - San Antonio, Viña del Mar - Quillota, Aconcagua, Metropolitano Norte, Metropolitano Occidente, Metropolitano Central, Metropolitano Oriente, Metropolitano Sur, Metropolitano Sur Oriente, O'Higgins, Maule; southern region provin [file 1471-2334-12-298-S1.doc]
