# Supplementary material for: The influence of climatic conditions on the transmission dynamics of the 2009 A/H1N1 influenza pandemic in Chile
Source: BMC Infect Dis. 2012 Nov 13;12:298. doi: 10.1186/1471-2334-12-298 (PMC3518181; doi:10.1186/1471-2334-12-298)
Supplement: Additional file 2 — Estimation of the reproduction number. [file 1471-2334-12-298-S2.pdf]

1 Estimation of the reproduction number

In the early stages of an epidemic, when the effect of increasing incidence on the depletion of susceptibles is small, the growth of the epidemic is exponential in nature, with rate r[1]-[4]. Assuming the classical SEIR (susceptible-infectious-recovered) transmission model, the reproduction number, $\mathcal{R}_0$, is determined from

$$\mathcal{R}_0 = \left(1 + \frac{r}{\gamma}\right)\left(1 + \frac{r}{\kappa}\right), \tag{1}$$

where $1/\kappa$ and $1/\gamma$ are the latent and infectious periods, respectively.

Given M incidence measurements, y_i^{data}, at time points, t_i, separated by Δt ($i = 1, ..., M$), the best fit exponential rise r is determined by minimizing the Poisson negative log likelihood[5]:

$$-\log \mathcal{L} = -\sum_{i=1}^{M} \left(y_i^{\text{data}} \log(y_i^{\text{pred}}) - y_i^{\text{pred}}\right), \tag{2}$$

where

$$y_i^{\text{pred}} = \frac{e^{rt_i}\left(e^{r\Delta t} - 1\right)}{\left(e^{rt_M} - e^{rt_1}\right)}. \tag{3}$$

The s standard deviation upper and lower limits on r are determined from the values of r that yield

$$-\log \mathcal{L}(r) = -\log \mathcal{L}_{\text{min}} + s^2/2, \tag{4}$$

where $-\log \mathcal{L}_{\text{min}}$ is the minimum value of $-\log \mathcal{L}$[5].

1.1 Determination of exponential growth phase

The standard deviation width of an epidemic curve consisting of N incidence measurements, y_i^{data}, at t_i different time points ($i = 1, ..., N$) is

$$\sigma_t = \sqrt{\frac{\sum_{i=1}^{N}(t_i - \bar{t})^2 y_i^{\text{data}}}{\sum_{i=1}^{N} y_i^{\text{data}}}}, \quad (5)$$

where

$$\bar{t} = \frac{\sum_{i=1}^{N} t_i y_i^{\text{data}}}{\sum_{i=1}^{N} y_i^{\text{data}}}. \quad (6)$$

In this analysis we select the exponential rise portion of the epidemic curve by identifying incidence data points at the beginning of the epidemic that are sufficiently many standard deviations away from the time of peak incidence (denoted by t_{peak}) that a fit of an exponential curve to simulated data in that region provides unbiased estimates of the true exponential rise. The exponential rise region is thus the region where $t_i < (t_{\text{peak}} - f\sigma_t)$.

In order to determine the optimal cut off value, f, we perform exponential rise fits to simulated data incidence curves from an SEIR (susceptible-exposed-infectious-recovered) model as explained below. Then we choose the value of f that provides unbiased estimates of the true exponential rise.

We carry out a simulation study to generate synthetic data incidence curves from the SEIR transmission model. That is, to estimate the time variation of the incidence, $y^{\text{pred}}(t)$, we use a compartmental model that simulates the number of susceptible (S), exposed (E), infectious (I), and recovered (R) individuals in the population using the

coupled deterministic ordinary non-linear differential equations ([1]):

$$\begin{aligned} S' &= -\beta SI/N \\ E' &= \beta SI/N - \kappa E \\ I' &= \kappa E - \gamma I \\ R' &= \gamma I, \end{aligned} \tag{7}$$

where $1/\kappa$ and $1/\gamma$ are the average latent and infectious periods, respectively, $\beta = \mathcal{R}_0\gamma$ is the transmission rate, and population size is given by $N = S + E + I + R$. We assume that $1/\gamma = 1.5$ days and $1/\kappa = 1.5$ days, which are within the range of mean estimates for the 2009 influenza pandemic [6, 7, 8, 9].

We simulate the incidence curve, $\sum_t y^{\text{pred}}(t)$, under various $\mathcal{R}_0$ hypotheses from 1.1 to 2.0 in steps of 0.1, and then scale $\sum_t y^{\text{pred}}(t)$ to simulate $1,000$ cases detected during the epidemic. We then obtain the simulated data epidemic incidence curve by random Poisson variation of the number of detected cases around the average within each time bin.

The exponential growth phase consists of data points at the beginning of the epidemic incidence curve that are at least f standard deviations away from the epidemic peak. Values of f between 0.5 to 2.0 in increments of 0.25 are examined, and the data simulation and fitting procedure is repeated 1000 times for each value of f. The optimal value of f is the one that is as low as possible, while still providing an unbiased estimate of the initial exponential growth rate, r. The estimated $\mathcal{R}_0$ is determined from the r estimate using Equation 1.

We found that $f >= 1.0$ provides estimates of the true $\mathcal{R}_0$ unbiased to within one

standard deviation, and within 5% of the true $\mathcal{R}_0$.

In this analysis, we thus determine the exponential rise portion of the epidemic curve by selecting points that are at least 1.0 standard deviation from the epidemic peak.

References

[1] Anderson, R. M. and May, R. M. (1991) *Infectious diseases of humans.* Oxford, UK: Oxford University Press

[2] Favier, C., *et al* (2006) *Early determination of the reproductive number for dengue in Brazil* Tropical Medicine and International Health, Vol 11, No 3: 332-340.

[3] Wallinga J, Lipsitch M (2007) *How generation intervals shape the relationship between growth rates and reproductive numbers.* Proc Biol Sci 274: 599-604.

[4] Chowell, G. *et al* (2007) *Comparative estimation of the reproduction number for pandemic influenza from daily case notification data.* J. R. Soc. Interface, Vol 4, No 12: 155-166.

[5] Cowan, G. (1998) *Statistical Data Analysis* Oxford Science Publications.

[6] Fraser C, Donnelly CA, Cauchemez S, Hanage WP, Van Kerkhove MD, et al. (2009) *Pandemic potential of a strain of influenza A (H1N1): early findings.* Science 324: 1557-1561.

[7] Yang Y, Sugimoto JD, Halloran ME, Basta NE, Chao DL, et al. (2009) *The transmissibility and control of pandemic influenza A (H1N1) virus.* Science 326: 729-733.

[8] Cauchemez S, Donnelly CA, Reed C, Ghani AC, Fraser C, et al. (2009) *Household transmission of 2009 pandemic influenza A (H1N1) virus in the United States.* N Engl J Med 361: 2619-2627.

[9] Cowling BJ, Chan KH, Fang VJ, Lau LL, So HC, et al. (2010) *Comparative epidemiology of pandemic and seasonal influenza A in households.* N Engl J Med 362: 2175-2184.
